# OCLSTM: Optimized convolutional and long short-term memory neural network model for protein secondary structure prediction

**DOI:** 10.1371/journal.pone.0245982

**Published:** 2021-02-03

**Authors:** Yawu Zhao, Yihui Liu

**Affiliations:** School of Computer Science and Technology, Qilu University of Technology (Shandong Academy of Sciences), Jinan, China; UMR-S1134, INSERM, Université Paris Diderot, INTS, FRANCE

## Abstract

Protein secondary structure prediction is extremely important for determining the spatial structure and function of proteins. In this paper, we apply an optimized convolutional neural network and long short-term memory neural network models to protein secondary structure prediction, which is called OCLSTM. We use an optimized convolutional neural network to extract local features between amino acid residues. Then use the bidirectional long short-term memory neural network to extract the remote interactions between the internal residues of the protein sequence to predict the protein structure. Experiments are performed on CASP10, CASP11, CASP12, CB513, and 25PDB datasets, and the good performance of 84.68%, 82.36%, 82.91%, 84.21% and 85.08% is achieved respectively. Experimental results show that the model can achieve better results.

## Introduction

Protein is the material basis of life activities, the basic organic matter that constitutes cells, and the main bearer of life activities. The structure of protein determines its function, so the prediction of protein structure has great research value. The structure of a protein mainly includes primary structure, secondary structure, tertiary structure and quaternary structure. The primary structure of a protein is the basic structure of the protein, and the amino acid sequence corresponds to the protein structure. Protein secondary structure is formed by folding based on protein primary structure. The tertiary structure of the protein is further coiled and folded based on the secondary structure, and the specific spatial structure formed by the maintenance of the secondary bond is called the tertiary structure of the protein. And so on, protein quaternary structure refers to a polymer structure formed by connecting multiple polypeptide chains with independent tertiary structure through non-covalent bonds. In the field of bioinformatics, protein secondary structure prediction is a very important and challenging problem [1], it is difficult to predict the spatial structure of a protein from a primary structure, so the prediction of protein secondary structure has been valued by many people.

At present, there are many basic methods to predict the protein secondary structure. For example, traditional machine learning methods: SVM [2], Bayesian algorithm [3]. In recent years, big data, deep learning methods, and other technologies have been widely used. Combined with deep learning models to predict the secondary structure of the protein has become a trend in research. Convolutional neural networks [4] have the characteristics of local perception, weight sharing, and downsampling. The convolutional neural network proposes that each neuron does not need to perceive all the pixels in the image, but only perceives the local pixels of the image, and then combines this local information at a higher layer to obtain all the characterization information of the image. The weight-sharing network structure reduces the complexity of the network model and the number of weights. The pooling layer does not perform any learning and is usually referred to as a form of nonlinear downsampling. The result of the merging process is to reduce the feature size and parameters to reduce the amount of calculation and increase the calculation speed. MUFOLD-SS [5] proposed a new deep neural network architecture, named the Deep inception-inside-inception (Deep3I) network for protein secondary structure prediction. Wavelets and convolutional neural networks [6] first used wavelets to extract features from the PSSM matrix and then input them to the convolutional neural network to further extract features. DeepCNF [7] used deep neural networks and conditional neural fields to predict for 3- and 8-state secondary structure. PSRM [8] utilized big data to train support vector machines [9]. This training uses protein length-based division and random subspaces of training data to train on various training targets. Compared with machine learning methods, convolutional neural networks can automatically extract local features of amino acid residues. For example, convolutional neural networks [7,10] and recurrent neural networks [11] have achieved remarkable results. In 1997, Hochreiter first proposed the Long Short-Term Memory (LSTM) [12], which is a special recurrent neural network (RNN). It can effectively solve the problem of gradient disappearance or gradient explosion of RNN and can learn long-distance dependencies. Guo et.al [13] fused asymmetric convolutional neural networks and long short-term memory neural network models and applies them to predict eight classes of the secondary structure of proteins. The paper [11] used long short-term memory (LSTM) bidirectional recurrent neural networks (BRNN), which can capture long-range interactions without using sliding windows. Paper [14] used bidirectional long and short-term memory neural networks to capture the long-distance correlation between amino acid residues for secondary structure prediction.

Most of the above models improve the structure of the deep network and add features to improve the prediction accuracy rate. However, the selection of network model parameters is based on manual adjustment, these models of parameters have not been optimized. To solve this problem, in this paper we used Bayesian optimization convolutional neural network was combined with long short-term memory neural network models for the prediction of protein secondary structure. The model combines the optimized convolutional neural network and BiLSTM neural network and used the protein feature matrix to predict the protein secondary structure. The optimized convolutional neural network can extract local features between complex amino acid residues in protein sequences. Besides, the BiLSTM neural network can further extract complex remote interactions between amino acids. Experimental results prove that the model in this paper can achieve better results.

## Materials and methods

### A. Model structure

**Fig 1:**
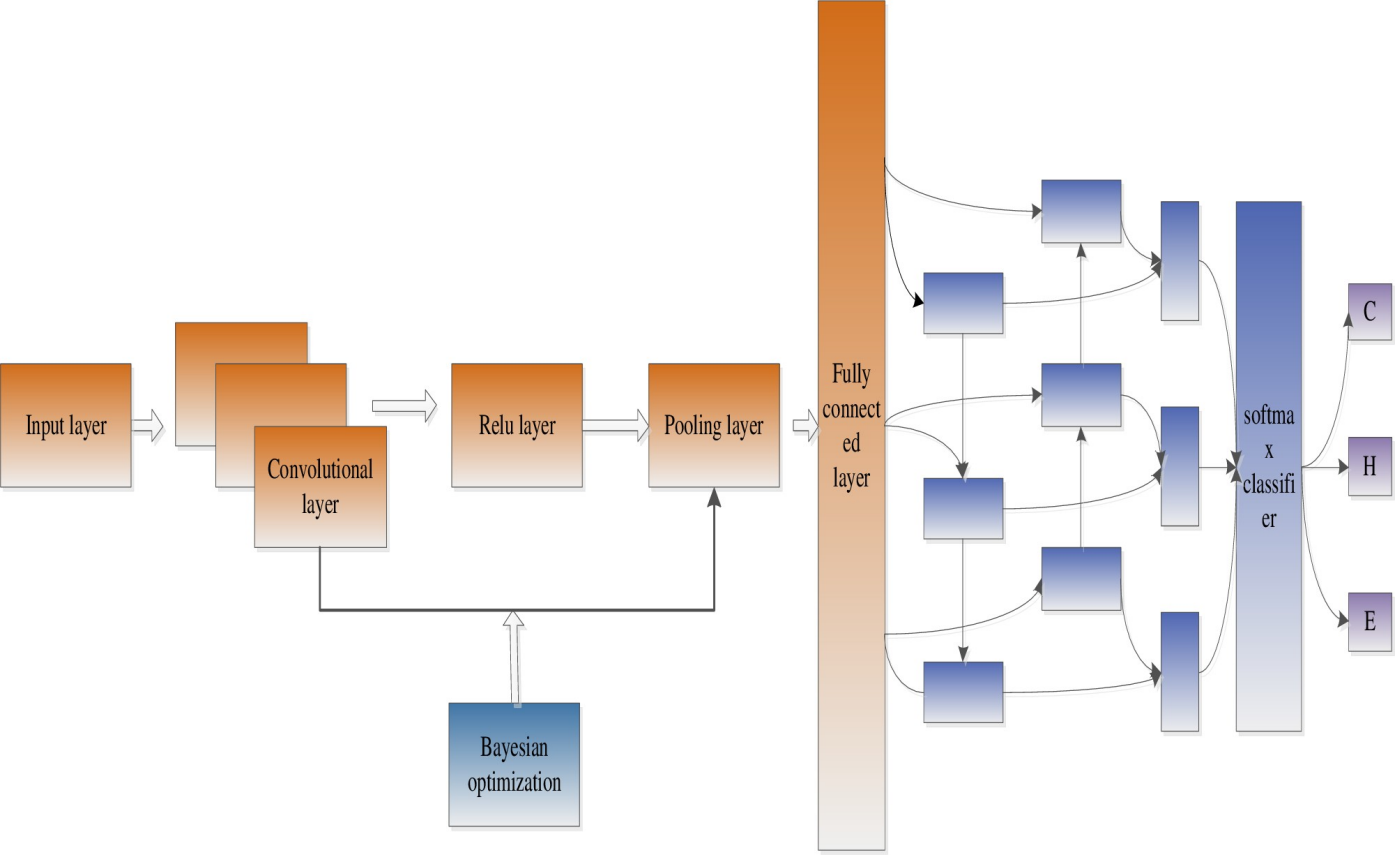
Model structure (Bayes optimizes the convolutional neural network and extracts local features, and BiLSTM extracts global features).

We proposed an optimized convolutional neural network and BiLSTM neural network model for protein secondary structure prediction. Firstly, the Bayesian algorithm is used to optimize the learning rate, network layers, gradient impulse, and regularization coefficients of the convolutional neural network. Through continuous iteration, the optimal network structure can be obtained. Secondly, extract the fully connected layer features of the convolutional neural network as the input of the BiLSTM neural network, and adjust the number of hidden unit layers. Fig 1 shows the model of this paper.

To get the best convolutional neural network structure, position-specific scoring matrix (PSSM) [15] represents the evolutionary information of acid amino sequence and can be used as a feature vector for predicting secondary structure. PSI-BLAST [16] software is used to calculate the position specific scoring matrix. In this paper, the PSSM evolution matrix is generated by multiple sequence alignment on the NR database using the PSI-BLAST program. The parameter of the PSI-BLAST program is set to a threshold of 0.001, and 3 iterations are performed to generate a 20xN matrix, where N is the length of the amino acid sequence and 20 represents the type of amino acid. The filter parameter is used to mask out the low complexity in the input sequence or the known repetitive sequence. There are two options, T and F. When we select "T" in this program, the program will shield simple repetitions and low-complexity sequences. Such as, when the sliding window is 13, to define the protein sequence as the center of the first sliding window, then it is necessary to make up 6 zeros before the first amino acid of the protein sequence and 6 zeros after the last amino acid of the protein sequence to cover all the residue, the processed PSSM matrix is used as the input to the convolutional neural network.

The widely used protein structure definition DSSP [17] contains eight class secondary structures, which are H (α-helix), B (β-turn), E (folded), G (3-helix), and I (5-helix), T (corner), S (curl), and L (ring). In this paper, G, H, I will be replaced by H, B, E will be replaced by E, and the rest will be replaced by C.

The experimental environment of this article is as follows: The cluster hardware is composed of 4 NF5280M5, 1 NF5288M5, and two Gigabit switches. NF5280M5 is equipped with Tesla V100 GPU (16G memory) computing card, NF5288M5 is equipped with Tesla V100 GPU (32G memory) computing card, and all nodes in the cluster are installed with Centos7.4 X64 standard system.

### B. Datasets

In this research, we selected seven public datasets: ASTRAL [18], CullPDB [19], CASP10 [20], CASP11 [21], CASP12 [22], CB513 [23] and 25PDB [24]. The ASTRAL dataset is version 2.03 released in 2013, it contains a total of 59514 proteins and contains more than 65% of the protein structure in the protein database. The CullPDB dataset contains 12,288 proteins. The CullPDB dataset was selected based on the percentage identity cutoff of 25%, the resolution cutoff of 3 angstroms, and the R-factor cutoff of 0.25. We removed the repeated protein sequences from the ASTRAL and CullPDB dataset and obtained 15,696 proteins as the training set.

Public datasets CASP10, CASP11, CB513, and 25PDB were used to test the model in this paper, and sequence identity is less than 25%. There are no duplicate protein sequences in the training and test dataset. The number of protein sequences is shown in Table 1.

**Table 1:** Number of proteins in the test datasets.

Test dataset	Number of proteins	Number of original proteins
CASP10	51	99
CASP11	36	81
CASP12	9	19
CB513	411	513
25PDB	999	1672

## OCLSTM

### A. Convolutional neural network

Convolutional neural networks [25] can extract local features between amino acid residues and improve the accuracy of protein secondary structure prediction. For the PSSM matrix, the data is divided in a sliding window manner as the input of the convolutional neural network. The convolutional layer is used to perform local feature extraction on the input data through local convolution and weight sharing. The input of each neuron in the convolutional layer comes from the neurons in a specific area of the previous layer feature map and the size of this specific area is determined by the convolution kernel. The process of convolution is to realize the convolution operation through the feature extraction filter "sliding" on the input matrix PSSM. Each region must be multiplied by the input matrix and weights, and then added with the offset parameter b_k_ to obtain the feature map. The feature map *c*^i^ is defined as follows:

$$C^{i}=(C_{1}^{i},C_{2}^{i},C_{3}^{i},\ldots ,C_{k}^{i},\ldots ,C_{N}^{i})$$ (1)

*C*_*i*_ is a convolution kernel group of the i-th layer, $C_{k}^{i}$ is a convolution kernel of the i-th layer, _N_ is the number of convolution kernels of the i-th layer. In the Bayesian optimization process, the number of convolution kernels is proportional to the depth of the network, so that networks of different depths have approximately the same number of parameters. In this paper, when the sliding window is 13 and 19, the depth of NetworkDepth is selected between [1,7] and the number of convolution kernels is 256/sqrt (NetworkDepth). For example, when the NetworkDepth is 4, the NetworkDepth parameter controls the depth of the network. The network has four parts, and each part has the same NetworkDepth convolutional layer. Therefore, the total number of convolutional layers is 4*NetworkDepth. In each layer, the number of convolution filters proportional to 256/sqrt(NetworkDepth) is used. $W_{h}^{i-1}$ is an area map generated by the input amino acid PSSM matrix and the convolution kernel from the previous layer. After the convolution of the i-th layer, the feature map $J_{k}^{i}$ can be obtained, which is defined as follows:

$$J_{k}^{i}=f\left(\sum_{h}W_{h}^{i-1}*C_{k}^{i}+b_{k}\right)$$ (2)

Variable *b*_*k*_ is the offset parameter, *f* is the activation function and the activation function is Relu. It is role is to perform non-linear operations on the output of the convolutional layer.

The pooling layer does not perform any learning, it is often referred to as a form of non-linear downsampling. The result of the pooling process is to reduce the feature dimensions and parameters to increase the calculation speed. It can also effectively reduce overfitting, and also have the characteristics of constant translation, which increases robustness.

Each neuron in the fully connected layer must be connected to the neurons in the previous layer, and the neurons in the fully connected layer are not connected to each other so that the local features extracted by the convolutional layer and the pooling layer can be synthesized to obtain global characteristics. The Softmax layer is the output layer, which is composed of three neurons. The output of this layer satisfies the following formula:

$$\sum_{j=1}^{3}P_{j}=1$$ (3)

Variable j represents the structures E, H and C of the protein.

### B. Bayesian optimization

In recent years, with the development of computer science and technology, the rise of various industries and fields has been born. The larger amounts of data generated by these industries also require more complex decision-making algorithms. For the above complex problems, Bayesian optimization algorithms [26] are an effective solution. The Bayesian optimization algorithm only needs a few objective function evaluations to obtain better results, and the Bayesian optimization algorithm has been widely used in games [27], recommendation systems [28], and navigation [29]. Due to the increasing number of protein sequences, applying the convolutional neural network model to the prediction of protein secondary structure, it takes a long time to adjust the hyperparameters of the convolutional neural network. Therefore, this paper uses a Bayesian optimization algorithm to optimize the convolutional neural network of hyperparameters.

The model proposed in this paper specifies the architecture of the convolutional neural network and the variables to be optimized, it includes learning rate, gradient impulse, regularization coefficient, and network layer and these variables are used as options for training algorithms. Create an objective function for the bayesian optimizer using training and validation data as inputs, and this function uses the variables to be optimized as input to train and verify the network. The CASP10 data set is used as the validation set.

The optimization of the hyperparameters of the convolutional neural network can be regarded as the optimization of the unknown black-box function. Bayesian optimization is to find the minimum value of the loss function f(*x*) on the bounded set D. It can construct a probability model for the function f(*x*) and use this model to judge how the set D evaluates the function. First, assume that the Gaussian kernel function is an optimized black-box function, and then choose an acquisition function to determine the next sampling point. Bayesian optimization of hyperparameters is Gaussian prior modeling of the loss function f(*x*) by hyperparameters.

$$L(m_{x},V_{h})=\sum_{(x_{i},y_{i})\in V_{h}}I(m_{x}(x_{i}),Y_{i})$$ (4)

Since the observations on the validation set have noisy, so add gaussian noise to each observation.

$$f(x)=L(m_{x},V_{h})+\theta$$ (5)

*m*_*x*_ is the model parameter generated by the convolutional neural network, including the learning rate, gradient impulse, and regularization coefficient. I(*m*_*x*_(*x*_*i*_),*Y*_*i*_) is objective function and $\theta ∼N\left(0,\;\sigma_{n}^{2}\right)$.

When using Gaussian process regression, there is no need to declare a specific function form. Any finite number of hyperparameters causes a multivariate gaussian distribution, which is determined by the covariance function K and the mean function *μ*_*x*_.

$$L∼GP(\mu_{x},K)$$ (6)

Where *K* = [*k*(x_*_,*x*_1_),*k*(x_*_,*x*_2_),…,*k*(x_*_,*x*_n_)]^*T*^, x_*_ are hyperparameters.

Suppose the input hyperparameters *X*_*i*_ = (*x*_1_, *x*_2_,…*x*_*n*_) gets the output *Y* = *L*(*x*_*i*_, *V*_*h*_) on the validation set. In each experiment, the Gaussian function evaluates f(*x*) based on the hyperparameter X_i_ and the validation set output Y and then the next set of hyperparameters is selected by the acquisition function. The predicted distribution of hyperparameter X_i_ is expressed as:

$$V[f(x_{i})]=k(x_{i},x_{i})-K_{*}^{T}{\left(K+\sigma_{n}^{2}I\right)}^{-1}K_{*}$$ (7)

In the process of Bayesian optimization of the parameters of the convolutional neural network, during each iteration, the collection function observes f(*x*), and then compares the next sampled hyperparameters to find the optimal solution. Expected Improvement [30] is defined as follows.

$$a(x|D)=E[\max(0,f_{x}-f_{best})]$$ (8)

Where *f*_*best*_ is the optimal solution based on the validation set.

To get the input of the BiLSTM neural network, we extract the output of the fully connected layer in the convolutional neural network. The protein sequence F represents as follows:

$$F=fc(W_{h}^{i-1}*J_{k}^{i}+b)$$ (9)

Where, the amino-acid sequence is represented as F: *F*_1_, *F*_2_,…,*F*_*N*-1_,*F*_*N*_.

### C. BiLSTM

**Fig 2:**
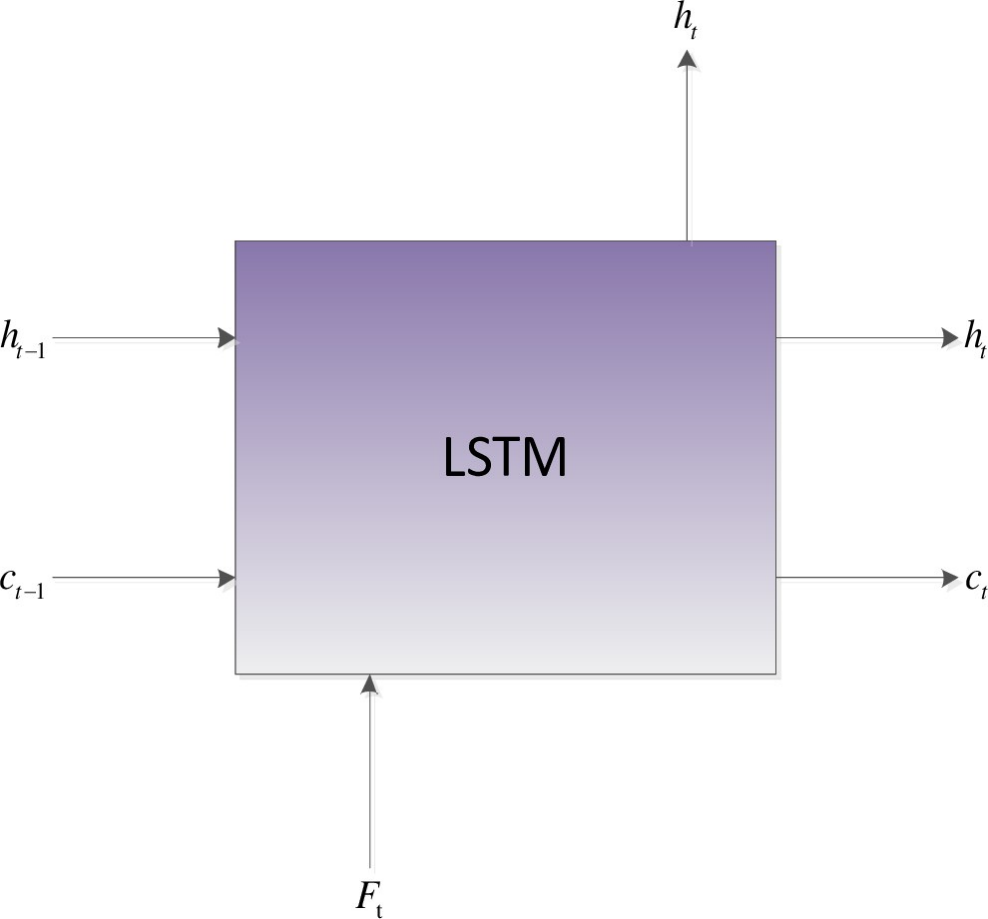
Internal architecture of the LSTM cell.

Compared with RNN, LSTM has designed the controller of the neural unit (Cell), which can judge whether the information is useful. In summary, the long-distance interdependencies [11,31] of amino acids are critical for protein secondary structure prediction. Therefore, local features extracted by the optimized convolutional neural network are sent to BiLSTM to obtain the long-distance dependencies of amino acids. The Cell control unit shows in Fig 2.

The control unit of the LSTM model consists of a memory unit that records the state and three gates (input gate *i*_*t*_, output gate *o*_*t*_, and forget gate *f*_*t*_). At time node t, the amino acid data enters the control unit for calculation. LSTM can choose to remember or forget certain information and control the output of information and pass this status information to the next time *t* + 1. The calculation method of each control information is as follows:

$$i_{t}=sigmoid(W_{i}h_{t-1}+W_{i}F_{t}+b_{i})$$ (10)

$$o_{t}=sigmoid(W_{o}h_{t-1}+W_{o}F_{t}+b_{o})$$ (11)

$$f_{t}=sigmoid(W_{f}h_{t-1}+W_{f}F_{t}+b_{f})$$ (12)

$$c_{t}=f_{t}⊗c_{t-1}+i_{t}⊗\tanh(W_{c}h_{t-1}+W_{c}F_{t}+b_{c})$$ (13)

$$h_{t}=o_{t}⊗\tanh(c_{t})$$ (14)

Where *f*_*t*_ is forgotten gate information at time t, *i*_*t*_ is input gate information at time t, *o*_*t*_ is output gate information at time t. *c*_*t*_ represents the update of the memory unit, *h*_*t*_ produces the current output, and decides which information is finally output. Moreover, W, b and ⊗ respectively represent weight matrix, bias value, and element-wise multiplication.

**Fig 3:**
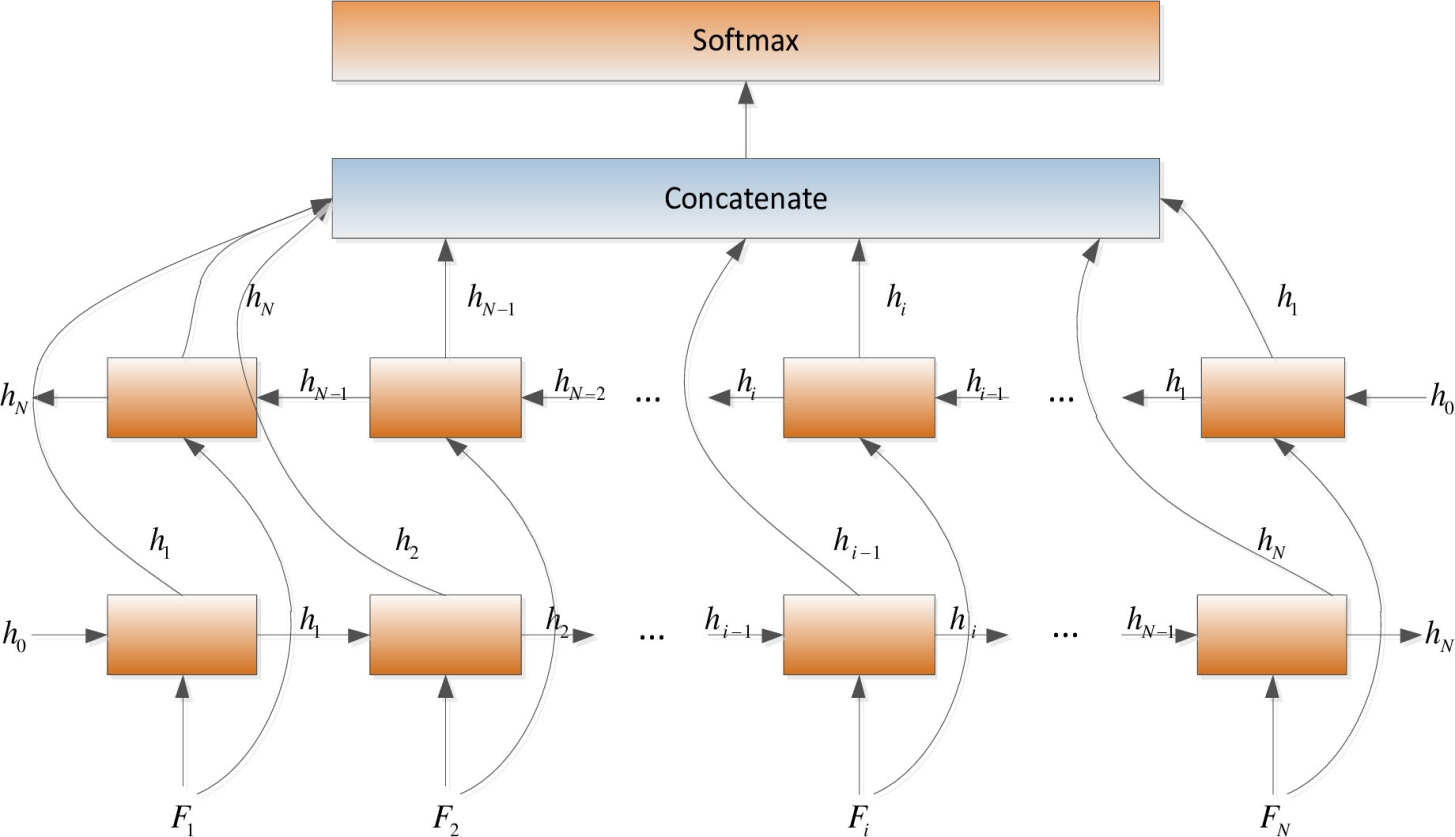
BiLSTM neural network structure.

In this paper, BiLSTM consists of a bidirectional LSTM neural network, as shown in Fig 3. The amino acid sequence was used as inputs to the forward and reverse LSTM networks to capture the long-distance dependence of amino acid residues. After the outputs of the two LSTM layers are combined, the softmax function is used for classification.

The inputs of the forward LSTM and backward LSTM in the BiLSTM model at time t are respectively:

$${\vec{h}}_{i}=\vec{L}(F_{i},{\vec{h}}_{i-1})$$ (15)

$${\overset{\leftarrow }{h}}_{i}=\overset{\leftarrow }{L}(F_{i},{\overset{\leftarrow }{h}}_{i-1})$$ (16)

Where, $\vec{h_{i}}$ is the output of the hidden state of the forward LSTM, $\overset{\leftarrow }{h_{i}}$ is the backward LSTM hidden state output. Combine the two as the hidden state output result *h*_*i*_ of BiLSTM.

## Results and discussion

As shown in Fig 1, we propose an optimized convolution and BiLSTM neural network model called OCLSTM. In the optimized convolution process, the NetworkDepth parameter controls the depth of the network. The network has four parts, and each part has the same NetworkDepth convolutional layer. Therefore, the total number of convolutional layers is 4*NetworkDepth. In each layer, the number of convolution filters proportional to 1/sqrt(NetworkDepth) is used. Therefore, for different part depths, the number of parameters and the amount of calculation required for each iteration are roughly the same. We used regularization to prevent overfitting. BiLSTM neural network can capture the long-distance dependence of the amino acid features extracted by the convolutional neural network.

By Bayesian optimization of the hyperparameters of the convolutional neural network, it can be found that the number of network layers, learning rate, gradient impulse, and regularization coefficient have a certain effect on the accuracy of the test dataset. The process of adjusting the hyperparameters show in Tables 2 and 3 (Network layers include: the input layer, convolutional layer, ReLU layer, pooling layer, fully connected layer, and softmax layer).

**Table 2:** Hyperparameters with sliding window of 13.

network layers	Learning rate	Gradient impulse	Regularization
10	0.0069	0.9359	2.15e-10
14	0.0013	0.8699	1.90e-04
18	0.0038	0.7562	5.62e-08
22	0.0027	0.9484	3.98e-06

**Table 3:** Hyperparameters with sliding window of 19.

network layers	Learning rate	Gradient impulse	Regularization
10	0.0035	0.7594	6.46e-05
14	0.0016	0.9455	7.11e-09
18	0.0024	0.6354	3.52e-07
22	0.0041	0.8720	4.52e-04

It is found through experiments that different hyperparameters have different accuracy rates. Different network structures can get different accuracy rates, as shown in Tables 4 and 5.

**Table 4:** Max pooling layer accuracy with a sliding window of 13.

Test dataset	10	14	18	22
CASP10	80.02	80.80	80.23	81.09
CASP11	77.49	79.98	78.25	78.30
CB513	78.29	82.42	80.53	80.05
25PDB	79.33	82.41	83.46	80.66

**Table 5:** Max polling layer accuracy with a sliding window of 19.

Test dataset	10	14	18	22
CASP10	79.97	80.09	80.02	81.36
CASP11	77.93	78.29	78.48	80.83
CB513	80.08	82.34	83.76	84.29
25PDB	81.45	83.40	83.80	84.80

The pooling layer includes max pooling and average pooling. As the hyperparameters of the convolutional neural network, the differences between them are: (1) the average pooling is to average the points in the area. (2) max-pooling is the maximum value of the output area. The accuracy rates in Tables 4 and 5 are the results obtained at the max-pooling layer. Tables 6 and 7 show the accuracy rate under average pooling.

**Table 6:** Average pooling layer accuracy with a sliding window of 13.

Test dataset	10	14	18	22
CASP10	79.92	80.23	79.81	80.47
CASP11	77.28	78.82	78.23	77.69
CB513	78.14	81.16	79.35	79.58
25PDB	79.29	81.54	82.77	80.02

**Table 7:** Average pooling layer accuracy with a sliding window of 19.

Test dataset	10	14	18	22
CASP10	79.51	79.48	79.68	80.95
CASP11	77.38	77.63	78.09	80.31
CB513	79.06	81.87	82.97	83.64
25PDB	79.87	83.04	83.42	84.18

The optimal network structure model can be obtained by comparing the results in Tables 4–7. When the sliding window is 19, the network model structure is 8 convolutional layers, the first four convolutional layers (convolution kernel size is 19, the number is 94), and the last four convolutional layers (convolution kernel size is 8, the number is 128), after each convolutional layer adds a ReLU layer. And after every four convolutional layers, the max-pooling layer size is 2*2.

In this paper, we obtain the optimal convolutional neural network structure through experiments. On this basis, local features of the convolutional neural network are extracted as the input of BiLSTM.

It can be from Table 8 that when the input feature dimension is 50, the CASP10 data set has the highest accuracy rate. We will adjust the number of neurons with a feature dimension of 50. LSMT1 is the number of neurons in the first layer, and LSTM2 is the number of neurons in the second layer. Q_3_ accuracy shows in Tables 9 and 10.

**Table 8:** Accuracy in different feature dimensions.

BiLSTM input dimension	CASP10
50	84.32
100	84.25
150	83.23
200	84.07
250	84.09
300	83.92
350	84.04
400	83.96
450	83.73
500	83.51
550	83.60
600	83.97

**Table 9:** Q3 accuracy the number of different neurons.

LSTM2	LSTM1				
	200	400	600	800	1000
200	83.88	83.94	84.46	83.86	84.31
400	84.29	83.97	81.75	83.87	83.53
600	83.93	84.15	83.42	84.04	84.30
800	84.07	83.99	84.19	83.84	84.53
1000	84.08	83.79	83.71	83.97	84.18

**Table 10:** The results on the test set of Q_3_ accuracy.

Dataset	Q_3_	Q_C_	Q_E_	Q_H_
CASP10	84.53	83.25	75.35	83.95
CASP11	80.61	77.63	74.63	86.82
CASP12	82.55	79.0	77.61	90.56
CB513	83.76	84.1	75.17	87.37
25PDB	84.89	83.24	79.28	90.14

In this paper, we make a comparative experiment to prove the effectiveness of the OCLSTM model. We use the unoptimized convolutional neural network combined with BiLSTM to predict protein secondary structure. The experimental results show in Table 11.

**Table 11:** Unoptimized convolution experiment results.

Dataset	Q_3_	Q_C_	Q_E_	Q_H_
CASP10	82.21	78.76	77.35	84.96
CASP11	79.41	80.21	78.36	82.67
CASP12	80.22	79.23	71.63	88.06
CB513	82.94	83.42	79.24	87.89
25PDB	84.31	82.4	80.35	89.72-

For the training dataset of 15696 proteins, one of 3 cross-validation experiments has 2540889 training samples and 1322362 test samples. The length of the sliding window is set to 13 and 19, to catch the long-range interaction of the amino acid sequence. Each experiment is running 3-fold cross-validation to 3 times. Table 12 shows the results based on CNN and OCLSTM are respectively.

**Table 12:** Q_3_ accuracy results of OCLSTM and CNN.

Sliding window	OCLSTM
13	79.78
19	80.09
	CNN
13	78.33
19	79.86

**Fig 4:**
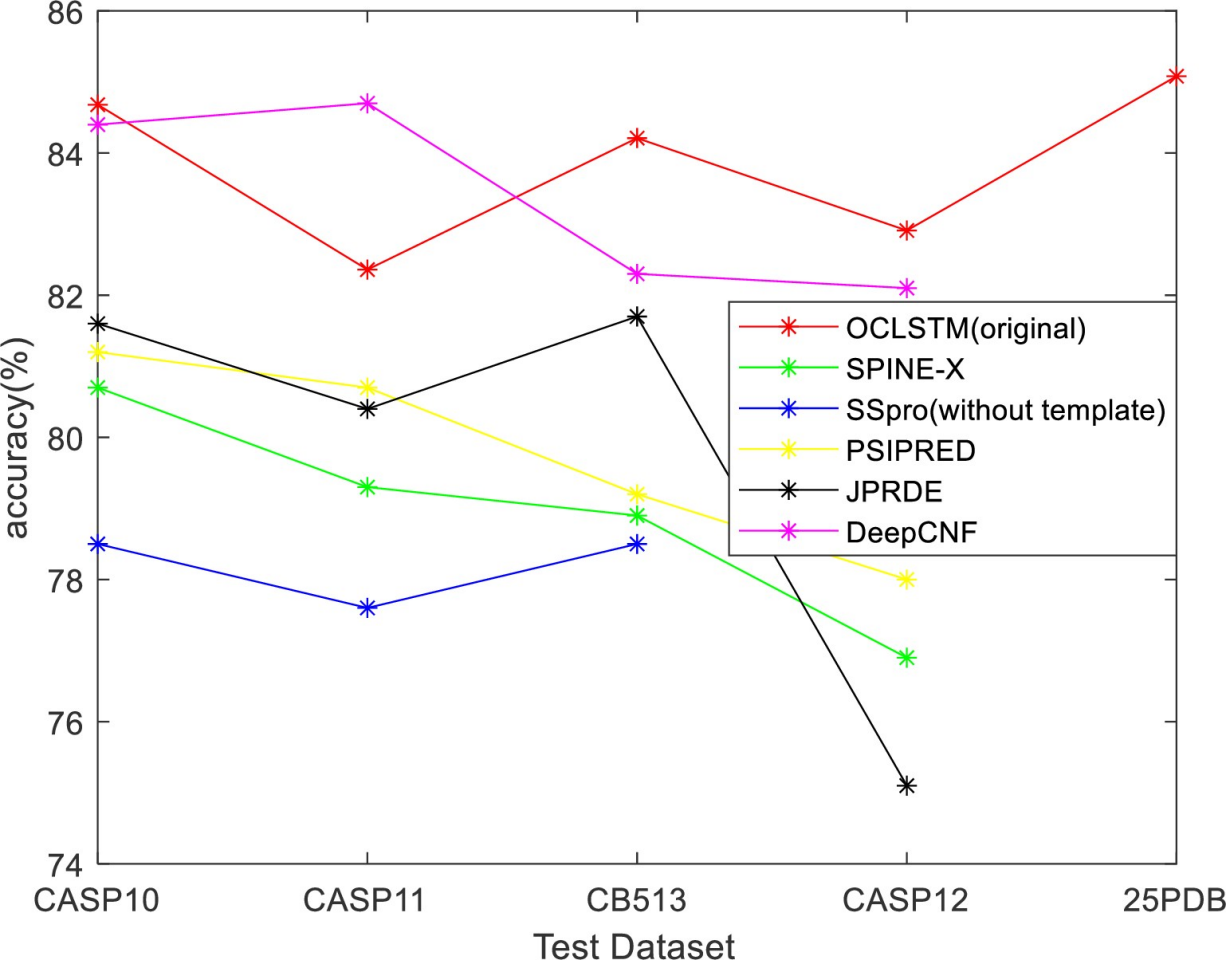
Q_3_ accuracy comparison between OCLSTM and other methods on CASP10, CASP11, CB513, CASP12 and 25PDB datasets.

In order to evaluate the accuracy of the model in this paper, four public test sets were used: CASP10, CASP11, CB513, and 25PDB. The model in this paper is compared with SPINE-X [32], SSpro [33], PSIPRED [34], JPRED [35], and DeepCNF [7] models. The accuracy of predicting the secondary structure of three types of proteins used as an index to evaluate the model in this paper. SPINE-X used a multi-step neural network, SSpro used a bidirectional naive recursive neural network, PSIPRED used a two-layer feedforward neural network, RaptorX-SS8 used a conditional neural field, and DeepCNF is a combination of a deep neural network and a conditional neural field. In Fig 4 and Table 13, the results of SPINE-X, SSpro, PSIPRED, JPRED, and DeepCNF on the test set are all taken from the paper [1,7]. We used the original test set and the duplicated test set to verify the OCLSTM model. The results show in Table 13.

**Table 13:** Q_3_ accuracy of the tested methods on CASP10, CASP11, CASP12, CB513and 25PDB datasets. (The results of SPINE-X, SSpro, PSIPRED, JPRED, and DeepCNF on the test set are all taken from PAPER [1,7]).

Methods	CSAP10	CASP11	CASP12	CB513	25PDB
SPINE-X	80.7	79.3	76.9	78.9	-
SSpro(without template)	78.6	77.6	-	78.5	-
PSIPRED	81.2	80.7	78.0	79.2	-
JPRED	81.6	80.4	75.1	81.7	-
DeepCNF	84.4	84.7	82.1	82.3	-
OCLSTM	84.53	80.61	82.55	83.76	84.89
OCLSTM(original)	84.68	82.36	82.91	84.21	85.08

## Conclusions

In bioinformatics, protein secondary structure prediction is a very important task. To better understand the relationship between protein sequences and structures, we propose an optimized convolutional neural network and long-term short-term memory neural network method, called OCLSTM. Compared with the latest method, our model achieved better results on three public test sets: CASP10, CASP11, and CB513. Convolutional neural networks can extract complex local features between amino acids, and BiLSTM can capture the correlation between distant amino acid residues. Experimental results show that OCLSTM can improve the Q_3_ accuracy of protein secondary structure prediction and have the ability of invariance of mutation, insertion, deletion of residue to some degree. The convolutional neural network uses the maximum pooling layer and selects the maximum value of the region as the feature. Therefore, when mutations, insertions, and deletions occur, there is a certain degree of mutation invariance. In future work, we will predict the Q_8_ accuracy of protein secondary structure and test our method on CASP, B513, and 25PDB datasets.

## Supporting information

S1 File (RAR)
